# Kinetic and Thermodynamic Studies on Synthesis of Mg-Doped LiMn_2_O_4_ Nanoparticles

**DOI:** 10.3390/nano10071409

**Published:** 2020-07-19

**Authors:** Aleksei Llusco, Mario Grageda, Svetlana Ushak

**Affiliations:** Departamento de Ingeniería Química y Procesos de Minerales and Center for Advanced Study of Lithium and Industrial Minerals (CELiMIN), Universidad de Antofagasta, Campus Coloso, Av Universidad de Antofagasta, 02800 Antofagasta, Chile; aleksei.llusco@uantof.cl (A.L.); svetlana.ushak@uantof.cl (S.U.)

**Keywords:** Lithium-ion batteries, LiMn_2_O_4_ nanoparticles, Mg-doped, kinetic and thermodynamic, thermogravimetric analysis, Pechini-type sol–gel process

## Abstract

In this work, a first study on kinetics and thermodynamics of thermal decomposition for synthesis of doped LiMn_2_O_4_ nanoparticles is presented. The effect of Mg doping concentration on thermal decomposition of synthesis precursors, prepared by ultrasound-assisted Pechini-type sol–gel process, and its significance on nucleation and growth of Mg-doped LiMn_2_O_4_ nanoparticles was studied through a method based on separation of multistage processes in single-stage reactions by deconvolution and transition state theory. Four zones of thermal decomposition were identified: Dehydration, polymeric matrix decomposition, carbonate decomposition and spinel formation, and spinel decomposition. Kinetic and thermodynamic analysis focused on the second zone. First-order Avrami-Erofeev equation was selected as reaction model representing the polymer matrix thermal decomposition. Kinetic and thermodynamic parameters revealed that Mg doping causes an increase in thermal inertia on conversion rate, and CO_2_ desorption was the limiting step for formation of thermodynamically stable spinel phases. Based on thermogravimetry experiments and the effect of Mg on thermal decomposition, an optimal two-stage heat treatment was determined for preparation of LiMg_x_Mn_2−x_O_4_ (x = 0.00, 0.02, 0.05, 0.10) nanocrystalline powders as promising cathode materials for lithium-ion batteries. Crystalline structure, morphology, and stoichiometry of synthesized powders were characterized by XRD, FE-SEM, and AAS, respectively.

## 1. Introduction

Lithium-ion batteries (LIBs) have been widely used in consumer electronics because of their remarkable characteristics, such as high energy and power density, low self-discharge rate, no memory effect, and long lifetime. In addition, LIBs have become the most attractive candidates as electrochemical storage systems for stationary applications, as well as power sources for sustainable electromobility and back-up supply applications [1,2,3,4].

Currently, there are five main technologies of LIBs used for portable applications, electric vehicles (EVs), and power supply systems: LiCoO_2_, LiNi_1−x−y_Mn_x_Co_y_O_2_, LiNi_0.8_Co_0.15_Al_0.05_O_2_, LiFePO_4_, and LiMn_2_O_4_ [5,6,7,8]. Among the existing cathode materials, LiMn_2_O_4_ (LMO) has been considered as one of the most viable cathodes for large-scale applications due to its several advantages such as easy preparation, low cost, abundance of raw materials, environmental friendliness, high cell voltage, and high rate capacity [9,10,11].

LMO crystallizes into a cubic crystal structure of $\mathrm{Fd}\bar{3}m$ space group with O ions at (32e) sites forming a compact, cubic, close-packed array. Tetrahedral (8a) sites are occupied by Li^+^ ions, while octahedral (16d) sites are occupied by Mn^3+^/Mn^4+^ ions. The remaining half of cationic octahedral sites in the structure are vacant (16c) sites [12]. Li^+^ ions occupying tetrahedral sites (8a) share common faces with four adjacent empty octahedral sites in position (16c). This lattice provides a three-dimensional structure of (16c)-(8a)-(16c) transport paths through which lithium ions diffuse during insertion/deinsertion reactions [13,14].

The LMO spinel can store a capacity of 148 mAh g^−1^, but unfortunately only 80% of Li^+^ ions can be deinserted from cathode material at a potential of 3–4.3 V vs. Li^+^/Li, providing a maximum practical capacity of 120 mAh g^−1^. About 20% of ions remain in the lattice and do not take part in insertion during cycling, resulting not only in low efficiency lithium utilization but also a potential safety problem when battery voltage exceeds cut-off voltage. In such abuse condition, remaining lithium ions in cathode material may be deinserted from the structure and deposited on anode surface, causing an internal short circuit in the battery [15,16].

Additionally, LMO presents severe problems of fading capacity during cycling, especially when temperature is above 55 °C. Reasons potentially responsible for poor electrochemical performance are (1) dissolution of Mn attributed to a disproportionation reaction of Mn^3+^ ion on the surface of particles and subsequent deposition of soluble Mn^2+^ ion in electrolyte on the negative electrode, which could lead to a decrease in active Mn^3+^ content and an increase in cell impedance; (2) Jahn-Teller distortion responsible for irreversible phase transition of the LMO spinel from a cubic phase to a tetragonal phase resulting in structural damage so that it blocks Li^+^ transport channels; and (3) high voltage-charge plateau and large amount of Mn^4+^ existing after full charge, which could accelerate electrolyte decomposition [17,18,19].

To solve these problems, several strategies were used by researchers: Partial replacement of Mn or O to stabilize host structure; surface modification to improve the interface; particle size, pore structure, and morphology to improve kinetic performance by reducing length of lithium ions’ and electrons’ transport paths [20]. Of all these approaches, (1) partial substitution of Mn^3+^ ions and (2) particle size control have proven to be effective in improving rate capability, cycle life, and discharge capacity at elevated temperatures of LMO [21].

Partial substitution of Mn^3+^ ions increases oxidation state of Mn in bulk LMO resulting in a strengthening of chemical bond between metal ions and oxide due to stronger chemical bonds of dopant, which prevent Mn^3+^ ions’ dissolution in electrolyte through dismutation reaction, and suppression of Jahn-Teller distortion [22]. Cation doping includes Li^+^, Ni^2+^, Zn^2+^, Mg^2+^, Al^3+^, Cr^3+^, Co^3+^, Ga^3+^, Ti^4+^, etc. [23]. Among potential doping elements, a transition nonmetal like Mg attracted a lot of attention due to its good electronic conductivity and its ability to stabilize the LMO host crystal structure [18,22,24,25,26,27,28,29,30,31] in addition to having many advantages, such as abundance, nontoxicity, low cost, and being lighter than transition metal elements.

On the other hand, particle size control at nanoscale has generated much attention for development of high-rate cathodes for LIBs, because nanometric or nanostructured materials facilitate rapid ion diffusion and electronic transport, increase electrode–electrolyte contact area, and improve electrolyte infiltration, allowing better power performance [32].

However, successful application of the above strategies for LMO spinel preparation depends largely on heat treatment conditions applied, i.e., temperature, atmosphere, and cooling rate, because these define the quality of powders through their physical characteristics, such as crystal morphology, exact composition, particle size, density, surface area, and their rechargeability into lithium ion battery cathodes. Therefore, particular attention must be placed in the determination of heat treatment parameters for synthesis of LMO spinels in order to obtain single-phase compounds with desired stoichiometry.

Pioneering research has studied in depth, by means of thermal analysis techniques, the decomposition of raw materials used in synthesis of LMO spinels by reaction in solid state and sol–gel, mainly, and thermal stability of spinel phase at high temperatures and different atmospheres [33,34,35,36,37,38,39]. However, only in [40] a dynamic study was carried out to determine kinetic parameters that were used as a theoretical basis to establish the optimal heat treatment conditions for synthesis of a stoichiometric LMO spinel by coprecipitation method, while no research was carried out on study of kinetics’ synthesis of doped LMO spinels.

Due to lack of theoretical information on the synthesis of doped LMO spinels, the main objective of this work was to develop a method of kinetic and thermodynamic analysis based on separation of multistage processes into one-stage reactions by deconvolution of the conversion rate curves obtained from thermogravimetry measurements. This allows for quantifying the effect of magnesium doping on thermal decomposition of precursors obtained by ultrasound-assisted, Pechini-type sol–gel process (PSG) at different concentrations of magnesium (x = 0.00, 0.02, 0.05, 0.10).

The method developed here comprises (1) deconvolution of conversion rate curves, (2) kinetic analysis of resulting individual curves to obtain a reaction model and kinetic parameters, (3) kinetic analysis of entire multistage process using the data obtained in each of the single-stage reactions, and (4) calculation of thermodynamic functions by means of transition state theory.

PSG is a solution technique used in preparation of materials that emerged as an alternative to conventional solid-state chemistry. This synthesis method is characterized by its ability to produce complex inorganic materials such as ternary and quaternary oxides at low process temperatures and shorter synthesis times. In addition, it offers several advantages, viz., low cost, homogeneous mixing at molecule level, low processing temperatures, use of an aqueous-based processing system, and proper control of stoichiometry, morphology, and particle size [41,42,43]. Application of the PSG method for preparation of LMO compounds was reported in previous investigations. Liu et al. [44] studied the influence of calcination temperature and effect of Ni doping on formation of LMO phase and determined that low calcination temperatures and high Ni ion concentration could favor formation of locally disordered crystallographic structures that would improve their cyclability and electrochemical performance. Han et al. [45] described effects of molar ratio of ethylene glycol (EG) to citric acid (CA) and calcination temperature on physicochemical and electrochemical properties of LMO. They found that at a temperature of 800 °C, the increase in EG/CA ratio resulted in an increase in homogeneity and surface area of spinel powders as well as their specific capacity and cyclability. Son et al. [46] reported the improvement of LMO electrochemical performance by partial replacement of Mn by Co, Cu, and Ga, which improved structural stability due to an increase in average Mn oxidation state, and surface modification by Ag particles’ coating, which in turn improved electrical conductivity and reduced surface overpotential of cathodic powders. In addition, they established that cyclability of cathodic materials was maintained regardless of synthesis temperature and particle size. Xiong et al. [47] studied LMO doping with multiple cations (Cu, Al, and Ti) and established that multiple doping was effective in improving retention capacity at temperatures of 25 °C to 50 °C, maintaining a high rate capability up to 12C (C represents the numerical value of rated capacity of a battery, in Ah) and reducing Mn dissolution. Amaral et al. [48] reported time and temperature effects of thermal treatment for Ga- and Al-doped LMO synthesis. They identified spinel phase formation at a temperature of 750 °C for a calcination time of 2 h and determined that the specific capacity of doped ones was lower than pure spinel. Here, PSG was useful to apply two improvement strategies, such as (1) particle size control at nanometer scale and (2) partial substitution of Mn^3+^ ions with Mg^2+^ ions, to stabilize host structure and improve electrochemical performance of the LMO spinel as a cathode material for LIBs.

Sources of Li and Mg, used as raw materials in the preparation of precursors, such as Li_2_CO_3_ (Albemarle) and Mg(OH)_2_ (obtained from the bischofite MgCl_2_*6H_2_O, which is an industrial waste derived from lithium production), respectively, came from the Salar de Atacama, Chile.

## 2. Materials and Methods

### 2.1. Synthesis of Mg-Doped LiMn_2_O_4_ Nanoparticles

Synthesis precursors were prepared by ultrasound-assisted, Pechini-type sol–gel method. The raw materials used as a source of metal ions were lithium carbonate Li_2_CO_3_ (battery grade ≥ 99.5%, Albemarle, Antofagasta, Chile), manganese acetate tetrahydrate Mn(CH_3_CO_2_)_2_*4H_2_O (≥99%, Sigma Aldrich, St. Louis, MO, United States), and magnesium hydroxide Mg(OH)_2_ (≥99%, CELIMIN, Antofagasta, Chile). Citric acid C_3_H_4_OH(COOH)_3_ (CA, Sigma Aldrich, St. Louis, MO, United States) and ethylene glycol HOC_2_H_4_OH (EG, Sigma Aldrich, St. Louis, MO, United States) were used as complexing and polymerization agents, respectively.

An initial solution was obtained by dissolving metal ion precursors in stoichiometric molar amounts in deionized water, taking into account four different concentrations of magnesium doping (x = 0.00, 0.02, 0.05, 0.10) and an excess of 5% lithium ions. Then, an equimolar solution of CA and EG in deionized water was added. The molar ratio between metal ions and CA was equal to 1:1. Both aqueous solutions were mixed by mechanical agitation at room temperature.

The resulting solution pH was adjusted to 6 by the addition of a 30 wt.% solution of ammonia NH_3_ in water. Increase in pH guaranteed deprotonation of two carboxylic groups from the CA (middle carboxylic group with p*K*_a1_ = 3.13 and terminal carboxylic group with p*K*_a2_ = 4.76; p*K*_a1_ and p*K*_a2_ are acid dissociation constants) facilitating the formation of stable chelate complexes between metal ions and CA, in addition to preventing segregation of metal ions as precipitates.

**Figure 1:**
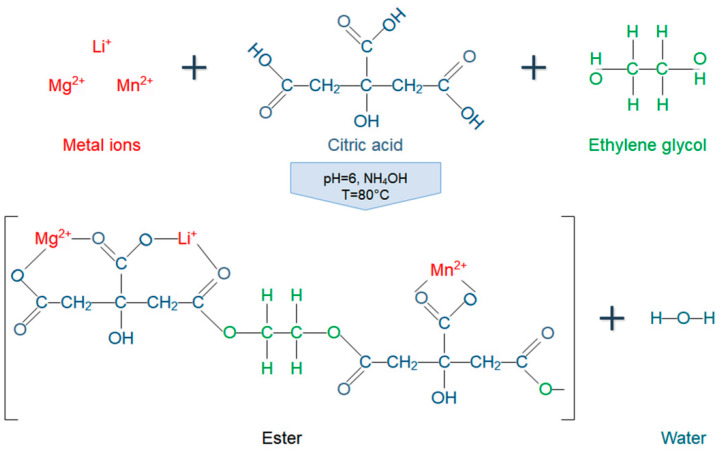
Schematic representation of condensation process between metal cations (Li^+^, Mg^2+^, and Mn^2+^), citric acid, and ethylene glycol from the initial stage of the Pechini-type sol–gel method.

The “sol” obtained was heated to 80 °C and then sonicated for 2.5 h at a frequency of 37 KHz and a power of 120 W by means of an ultrasonic bath (Elmasonic P 30H, Singen, Germany). Simultaneously, continuous mixing was maintained on the “sol” through a mechanical agitator at a rotation speed of 220 rpm. Cavitation was used to catalyze the condensation reaction between metal citrate complexes and EG. Condensation process is schematically represented in Figure 1.

After the mixing process, the “sol” was heated at a constant temperature of 120 °C by means of a heating plate (IKA C-MAG HS 10 digital, Staufen, Germany). Then, viscosity of the “sol” increased dramatically due to the polyesterification process that resulted in formation of a “gel”, which immobilizes metal complexes in a rigid organic polymer network.

The “gel” obtained was dried in an oven (Thermo Scientific Thermolyne F47900, Waltham, MA, United States) at a temperature of 170 °C for 18 h resulting in formation of a rigid and fragile porous structure or “xerogel”. The “xerogel” or synthesis precursor obtained was ground in an agate mortar for subsequent thermogravimetric (TG) analysis. Precursors with magnesium concentrations of 0.00, 0.02, 0.05, and 0.10 were identified as SP, SPMg-1, SPMg-2, and SPMg-3, respectively.

A two-stage heat treatment was programmed in a muffle furnace (Nabertherm L 40/12/B410, Lilienthal, Germany), based on results obtained from TG analysis, to obtain LiMg_x_Mn_2−x_O_4_ (x = 0.00, 0.02, 0.05, 0.10) nanocrystalline cathode powders. At the first stage, synthesis precursors were calcined in air at 500 °C for 4 h and at the second stage, spinel oxides were sintered in air at 750 °C for 12 h.

Obtained cathode material powders were identified as LMO, LMOMg-1, LMOMg-2, and LMOMg-3 for the magnesium doping concentrations of 0.00, 0.02, 0.05 and 0.10, respectively.

### 2.2. Materials’ Characterization

Thermogravimetric analysis (TG, NETZSCH STA 449 F3 Jupiter, Selb, Germany) was used to examine the thermal decomposition of synthesis precursors. Samples’ mass was recorded at a heating rate of 10 °C min^−1^ from room temperature 25 °C to 950 °C in a dynamic atmosphere of extra pure synthetic air.

A 4 M HCl solution was used to digest spinel powder in solution with a solid-to-liquid ratio of 1 g: 50 mL, and then the lithium, magnesium, and manganese contents of cathode materials were determined by atomic absorption spectrometry (AAS, VARIAN SpectrAA-220 FS—Agilent, Santa Clara, CA, United States) using air/acetylene flame. Powder X-ray diffraction (XRD, Bruker D8 Advance-A25, Billerica, MA, United States) was used to identify the crystalline phase of prepared materials. The diffraction patterns were recorded using a radiation source from CuKα (λ = 1.5406 Å) over a range of 2θ of 2–70° with a step size of 0.02°. On the basis of the X-ray diffraction data, structural parameters of synthetized spinels were refined using the Rietveld method as implemented in the FullProf suite.

Particle size and morphology of powder samples were studied by field emission scanning electron microscopy (FE-SEM, Hitachi SU5000, Tokyo, Japan).

### 2.3. Kinetic Principle of Solid-State Reactions

Kinetics of a thermally stimulated solid-state reaction are defined through the conversion rate $\;\frac{d\alpha }{\mathrm{dt}}$, which is described by two independent functions: k(T) represents the influence of temperature and f(α)  represents the influence of conversion [36,37,38], as shown in Equation (1)

(1) $$\frac{d\alpha }{\mathrm{dt}}=k\left(T\right)f\left(\alpha \right)$$

where α is the degree of conversion of reaction from 0 to 1 as process proceeds from start to finish, T is the absolute temperature (K), k(T) is a temperature-dependent rate constant (s^−1^), and f(α) is the differential function of conversion or reaction model that describes the rate-limiting stage mechanism of different types of solid-state reactions.

Reaction rate dependence on temperature k(T) was parameterized by Arrhenius’ law [49,50]:

(2) $$k\left(T\right)=A\;\exp\left(-\frac{E_{a}}{\mathrm{RT}}\right)$$

where A is the pre-exponential factor (s^−1^), $E_{a}$ is the activation energy (kJ mol^−1^), R is the universal gas constant (8.314 J mol^−1^ K^−1^), and T is the absolute temperature.

The Sestak-Berggren (SB) equation [51,52] was applied to determine reaction models f(α) that describe the behavior of decomposition kinetics of synthesis precursors.

(3) $$f\left(\alpha \right)={\left(1-\alpha \right)}^{n}\alpha^{m}{\left[-\ln\left(1-\alpha \right)\right]}^{p}$$

where n, m, and p are kinetic exponents. The appropriate combination of n, m, and p allows numerous mathematical descriptions of different empirical kinetic models to be expressed. $\;\mathrm{The}\;{\left(1-\alpha \right)}^{n}$, $\alpha^{m}$, and ${\left[-\ln\left(1-\alpha \right)\right]}^{p}$ represent three different mechanisms of interface reaction, diffusion, and nucleation, respectively. Different SB(n,m,p) models commonly used to describe kinetics of solid-state reactions can be found in literature [53,54].

Under non-isothermal heating conditions, linear change in temperature over time is represented by [55]:

(4) $$\beta =\frac{\mathrm{dT}}{\mathrm{dt}}=\mathrm{constant}$$

where β is the heating rate.

Entering Equations (2) to (4) in Equation (1), the kinetic equation is obtained under a non-isothermal linear heating program:

(5) $$\frac{d\alpha }{\mathrm{dT}}=\frac{A\;}{\beta }\exp\left(-\frac{E_{a}}{\mathrm{RT}}\right){\left(1-\alpha \right)}^{n}\alpha^{m}{\left[-\ln\left(1-\alpha \right)\right]}^{p}.$$

### 2.4. Deconvolution Function

The Fraser-Suzuki equation [56] (asymmetric Gaussian equation) was used as fitting function for the deconvolution process of conversion rate curves representing complex solid-state reactions as an overlap of individual processes.

(6) $$Y=a_{0}\exp\left[-\ln2{\left[\frac{\ln\left(1+2a_{3}\frac{X-a_{1}}{a_{2}}\right)}{a_{3}}\right]}^{2}\right]$$

where $a_{0}$, $a_{1}$, $a_{2}$, and $a_{3}$ are the amplitude, position, half-width, and asymmetry of the curve, respectively.

## 3. Results and Discussion

### 3.1. Thermogravimetric Data Analysis

Initially, experimental data of thermogravimetry (TG) and its derivatives (DTG) were normalized using Equations (7) and (8), respectively, in order to standardize mass-loss curves of precursors with different magnesium concentrations. Normalized TG was represented by M, where m is the mass in time (t) and $m_{o}$ the initial mass at 25 °C. Normalized DTG was represented by $\frac{\mathrm{dM}}{\mathrm{dt}}$, where $\frac{\mathrm{dm}}{\mathrm{dt}}$ corresponds to experimental DTG in mg s^−1^:

(7) $$M=\frac{m}{m_{o}}\cdot 100\%$$

(8) $$\frac{\mathrm{dM}}{\mathrm{dt}}=\frac{\mathrm{dm}}{\mathrm{dt}}\frac{1}{m_{o}}\cdot 100\%=\mathrm{DTG}\frac{1}{m_{o}}\cdot 100\%.$$

To perform kinetic analysis, experimental conversion $\alpha_{\exp}$ [50,57,58] and conversion rate ${\left(\frac{d\alpha }{\mathrm{dt}}\right)}_{\exp}$ through Equations (9) and (10) were determined, respectively, where $M_{o}$, M, and $M_{f}$ are standardized masses at the beginning, at time t, and at reaction end, respectively.

(9) $$\alpha_{\exp}=\frac{M_{o}-M}{M_{o}-M_{f}}$$

(10) $${\left(\frac{d\alpha }{\mathrm{dt}}\right)}_{\exp}=-\frac{\mathrm{dM}}{\mathrm{dt}}\frac{1}{\left(M_{o}-M_{f}\right)}$$

### 3.2. Thermal Decomposition of Synthesis Precursors

**Figure 2:**
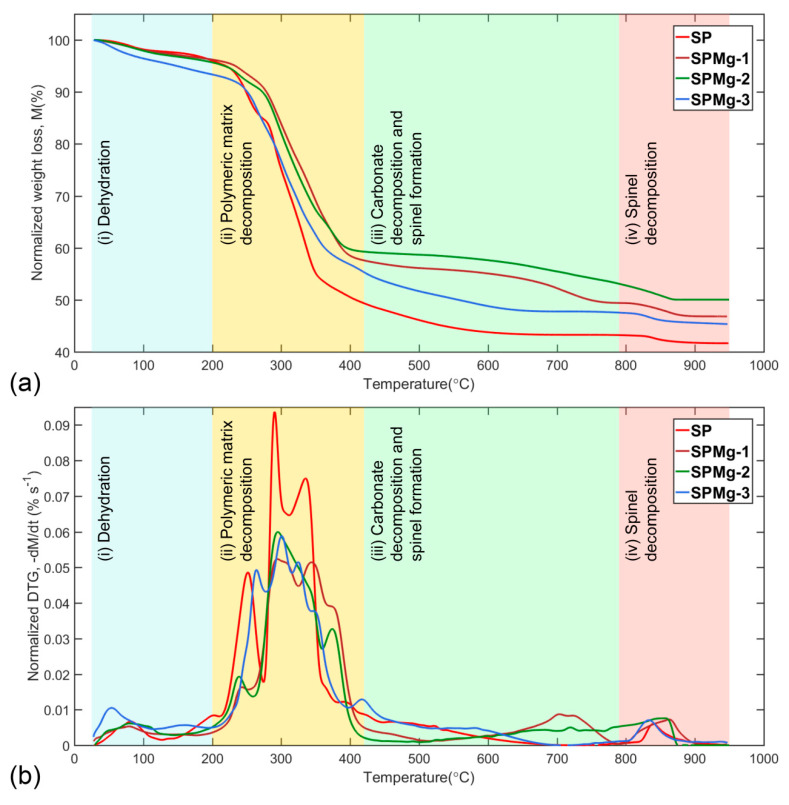
Normalized curves of (**a**) mass loss, M, and (**b**) DTG, as a function of temperature, of thermal decomposition of synthesis precursors divided into four zones: (1) Dehydration, (2) polymeric matrix decomposition, (3) carbonate decomposition and spinel formation, and (4) spinel decomposition.

Considering different magnesium concentrations used as doping agent, the thermal decomposition of synthesis precursors, at a heating rate of 10 °C min^−1^ in a temperature range of 25 °C to 950 °C, was divided into four roughly separate zones: (1) Dehydration, (2) polymeric matrix decomposition, (3) carbonate decomposition and spinel formation, and (4) spinel decomposition. Figure 2a,b presents normalized mass-loss curves (M) and its derivative ($\frac{\mathrm{dM}}{\mathrm{dt}}$) as a function of temperature according to Equations (1) and (2), respectively.

Dehydration: The first zone (25 °C to 200 °C) is related to the dehydration reaction, where the mass loss of the residual and adsorbed water of the synthesis precursors was recorded in a range of 2.19% to 6.45% in normalized TG curves (Figure 2a), and was observed as a small and wide peak in normalized DTG curves (Figure 2b) in a mass-loss rate range of 0.53 × 10^−2^% s^−1^ to 1.05 × 10^−2^% s^−1^ (Table 1). A steeper mass loss observed for the SPMg-3 precursor was related to a decrease in precursor density. This was due to the fact that high Mg concentration decreases precursor density, favoring formation of a more porous structure than that which could occlude a greater quantity of volatiles during gel-drying process.

Decomposition of polymeric matrix: The second zone (200 °C to 420 °C) is mainly associated with removal of the organic part of synthesis precursors, which contains the metal ions in a homogeneous composition in a polymeric network with a rigid structure.

In this zone, synthesis precursors record the highest mass loss in normalized TG curves of Figure 2a, between 36.61% and 46.02%, as a result of thermal decomposition process of the polymeric matrix that takes place through complex solid-state reactions and whose mass-loss rates overlap, as shown in Figure 2b, with maximum values of 5.24 × 10^−2^% s^−1^ to 9.37 × 10^−2^% s^−1^ (Table 1).

Thermal decomposition of polymeric matrix is divided into two parts. The first part comprises only endothermic transformations in dry air such as evolution of NH_3_ (Equation (11)) which removes the concentration of NH^4+^ species that are part of metal complex [59], and decomposition of citrate anion through evolution of H_2_O (Equation (12)) and CO_2_ (Equation (13)) [60,61,62], while the second part comprises exothermic transformations in dry air such as thermo-oxidative decomposition of organic composition (Equations (14)–(16)) [63].

- Ammonia evolution reaction:
  (11) $$2{\mathrm{NH}}_{4}^{+}+\frac{1}{2}O_{2}+\mathrm{heat}\to 2{\mathrm{NH}}_{3}+H_{2}O$$
- Dehydroxylation reaction:
  (12) $$\begin{array}{c} {\mathrm{Li}}_{2}{\left({\mathrm{Mg}}_{x}{\mathrm{Mn}}_{2-x}\right)}_{2}{\left(C_{6}H_{6}O_{7}\right)}_{5}+\mathrm{heat}\to {\mathrm{Li}}_{2}{\left({\mathrm{Mg}}_{x}{\mathrm{Mn}}_{2-x}\right)}_{2}{\left(C_{6}H_{4}O_{6}\right)}_{5}+5H_{2}O\\ \mathrm{citrate complex}\;\;\mathrm{aconitate complex} \end{array}$$
- Decarboxylation reaction:
  (13) $$\begin{array}{c} {\mathrm{Li}}_{2}{\left({\mathrm{Mg}}_{x}{\mathrm{Mn}}_{2-x}\right)}_{2}{\left(C_{6}H_{4}O_{6}\right)}_{5}+\mathrm{heat}\to {\mathrm{Li}}_{2}{\left({\mathrm{Mg}}_{x}{\mathrm{Mn}}_{2-x}\right)}_{2}{\left(C_{5}H_{4}O_{4}\right)}_{5}+5{\mathrm{CO}}_{2}\\ \mathrm{aconitate complex}\;\;\mathrm{itaconate/citraconate complex} \end{array}$$
- Thermo-oxidative reaction of organic composition:
  (14) $$2{\mathrm{Li}}_{2}\left(C_{5}H_{4}O_{4}\right)+9O_{2}\to 2{\mathrm{Li}}_{2}O+10{\mathrm{CO}}_{2}+4H_{2}O+\mathrm{heat}$$
  (15) $$2\mathrm{Mg}\left(C_{5}H_{4}O_{4}\right)+9O_{2}\to 2\mathrm{MgO}+10{\mathrm{CO}}_{2}+4H_{2}O+\mathrm{heat}$$
  (16) $$4\mathrm{Mn}\left(C_{5}H_{4}O_{4}\right)+19O_{2}\to 2{\mathrm{Mn}}_{2}O_{3}+20{\mathrm{CO}}_{2}+8H_{2}O+\mathrm{heat}$$

As a consequence of dehydroxylation and subsequent decarboxylation of the metal complex, Li^+^, Mg^2+^, and Mn^2+^ ions are uncoordinated from their ligands and are available to form mixed oxide with spinel structure after organic composition combustion of precursors. However, an intermediate phase composed of oxides and carbonates was obtained instead of a pure spinel phase.

Formation of intermediate carbonates occurs as a result of chemisorption of CO_2_, a volatile product from decomposition of organics, by a fraction of metal oxides. This behavior was clearly observed in Figure 2b as a long tail after the last peak of polymeric matrix decomposition [64,65,66].

Carbonate decomposition and spinel formation: The third zone (420 °C to 790 °C) comprises decomposition of the intermediate phase carbonates to obtain a stable oxide phase with spinel structure.

With increasing temperature, additional heat applied to samples induces desorption of CO_2_ from metal oxides and formation of target phase as a solid-state solution of lithium, magnesium, and manganese oxides (Equation (17)). Therefore, CO_2_ removal from precursors was the only limiting step for formation of a thermodynamically stable spinel phase in this zone.

Manganese spinel formation as solid-state solution:

(17) $$\frac{\left(1+y\right)}{2}{\mathrm{Li}}_{2}O+\mathrm{xMgO}+\frac{\left(1+y-2x\right)}{2}{\mathrm{Mn}}_{2}O_{3}+\left(1-y+x\right){\mathrm{MnO}}_{2}\to {\mathrm{Li}}_{1+y}{\mathrm{Mg}}_{x}{\mathrm{Mn}}_{2-x}O_{4}$$

However, only undoped magnesium precursor formed a pure and stable cubic spinel phase between 700 °C and 800 °C, a temperature range in which mass of formed phase remained constant (Figure 2a) with mass-loss rate equal to 0 (Figure 2b).

In contrast, magnesium-doped precursors did not form a pure spinel phase and maintained a mixed composition of oxides and carbonates with a CO_2_ mass retention of 7.09%, 11.01%, and 2.36% (Figure 2a) for magnesium concentrations of 0.02, 0.05, and 0.1, respectively, compared to pure phase of nonmagnesium precursor in the same temperature range.

Taking into account the decomposition order of carbonates with temperature ${\mathrm{MnCO}}_{3}\sim 515\;{}^{\circ}C—680\;{}^{\circ}C<{\mathrm{MgCO}}_{3}\sim 620\;{}^{\circ}C—650\;{}^{\circ}C<{\mathrm{Li}}_{2}{\mathrm{CO}}_{3}\sim 700\;{}^{\circ}C—1100\;{}^{\circ}C$ [67,68,69,70], stable (SPMg-1 and SPMg-3) and intermediate (SPMg-2) phases at the end of this zone would be mainly composed of a mixed metal oxide with an ${\mathrm{Li}}_{2}{\mathrm{CO}}_{3}$ excess.

Spinel decomposition: The fourth zone (790 °C to 950 °C) is mainly associated with oxygen loss from spinel structure, which recorded a mass loss of 1.61% to 3.69% on normalized TG curves (Figure 2a) and was observed as a small and wide peak (for magnesium-doped precursors due to superimposition of ${\mathrm{Li}}_{2}{\mathrm{CO}}_{3}$ decomposition rate) on normalized DTG curves (Figure 2b) in a mass-loss rate range of 0.6 × 10^−2^% s^−1^ to 0.76 × 10^−2^% s^−1^ (Table 1).

Continuous mass decrease due to gradual loss of oxygen from spinel phase promotes diffusion of lithium to particles’ surface, where disproportionation reaction takes place resulting in (1) formation of a stable rock-salt phase ${\mathrm{Li}}_{2}{\mathrm{MnO}}_{3}$ and (2) change of crystalline symmetry of spinel from cubic to tetragonal due to Mn^3+^ concentration increase, which decreases average oxidation state of manganese below 3.5 [36,71,72,73]. Disproportionation reaction of spinel phase is represented by the following equation:

(18) $${\mathrm{LiMn}}_{2}O_{4}\to {\mathrm{Li}}_{1-2\delta }{\mathrm{Mn}}_{2-\delta }O_{4-3\delta -\;\delta \;\prime }+{\delta \mathrm{Li}}_{2}{\mathrm{MnO}}_{3}+\frac{\;\delta \;\prime }{2}O_{2}$$

With increasing temperature, a higher oxygen removal causes a new phase transformation as a result of the reaction between manganese-rich spinel phase and ${\mathrm{Li}}_{2}{\mathrm{MnO}}_{3}$ phase to produce ${\mathrm{LiMnO}}_{2}$ [71,72] according to the following equation:

(19) $${\mathrm{LiMn}}_{2}O_{4}+{\mathrm{Li}}_{2}{\mathrm{MnO}}_{3}\to 3{\mathrm{LiMnO}}_{2}+\frac{1}{2}O_{2}$$

The percentages and maximum rates of mass loss of each of the decomposition zones of the synthesis precursors are detailed in Table 1, according to the magnesium doping concentrations.

### 3.3. Determination of Thermal Decomposition Kinetic Parameters of Polymeric Matrix

**Figure 3:**
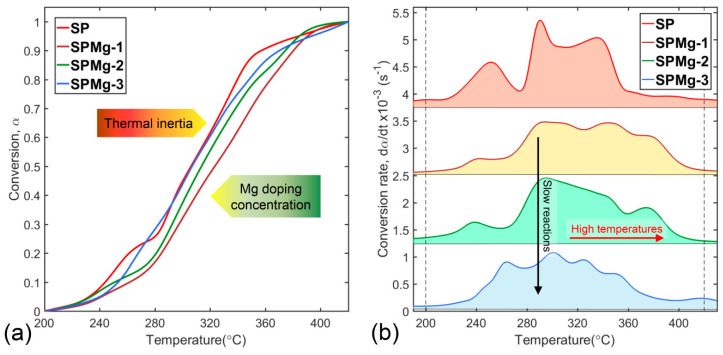
Curves of (**a**) conversion and (**b**) conversion rates’ evolution of synthesis precursors with different magnesium doping concentrations.

Analysis of decomposition kinetics of synthesis precursors was focused on the second zone, because decomposition of polymeric matrix encompasses fundamental transformation processes that initiate the formation of a thermodynamically stable spinel phase after removal of organic composition. In this second zone, synthesis precursors’ conversion was affected by magnesium concentration causing a displacement of conversion curves at higher temperatures compared to nondoped SP precursor as shown in Figure 3a. Displacement observed in conversion of SPMg-1, SPMg-2, and SPMg-3 precursors was inversely proportional to magnesium concentration and would be related to a decrease in thermal conductivity of samples due to an increase in thermal inertia.

As explained in Section 3.2, continuous availability of CO_2_ during polymeric matrix decomposition, first from decarboxylation reaction (Equation (13)) and secondly from organics’ combustion (Equations (14)–(16)), promoted carbonates’ formation as secondary phases, which would be the cause of increase in thermal inertia due to the fact that they require more energy to desorb CO_2_ and form a pure and stable spinel phase. Taking into account that CO_2_ amount released during thermal decomposition was equivalent for all precursors, the SPMg-1 precursor with lower concentration of magnesium showed a higher propensity to CO_2_ sorption and carbonates’ formation as secondary phases compared to the SPMg-3 precursor with higher concentration of magnesium. Therefore, a higher concentration of magnesium doping in precursors would reduce secondary phases’ formation and, consequently, thermal inertia.

Thermal inertia effects on multiple stages of polymeric matrix decomposition are shown in more detail in Figure 3b where peak positions of conversion rates of the SPMg-1, SPMg-2, and SPMg-3 precursors, compared to nondoped SP precursor, experienced a decrease in height and a shift toward higher temperatures in inverse proportion to magnesium doping concentration. This behavior revealed that magnesium incorporation decreases conversion speeds (slow reactions) and causes a delay in decomposition process, which is related to a higher energy consumption (high temperatures) to complete conversion.

To quantify the effect of magnesium doping concentration on polymeric matrix thermal decomposition, complex solid-state reactions represented by conversion rate curves (Figure 3b) were separated into five single-stage reactions using the deconvolution function of Equation (6).

Kinetic analysis of resulting individual curves, as independent reaction stages, was carried out using the linear form of Equation (5) [74]:

(20) $$\ln\left(\beta \frac{d\alpha }{\mathrm{dT}}\right)=\ln\left(A\right)-\frac{E_{a}}{\mathrm{RT}}+\mathrm{nln}\left(1-\alpha \right)+\mathrm{mln}\alpha +\mathrm{pln}\left[-\ln\left(1-\alpha \right)\right]$$

Reaction model (f(α)) and the approximate Arrhenius parameters ($E_{a}$ and A) for each individual reaction were obtained from Equation (20) by multiple linear regression and results were obtained as shown in Table 2.

For kinetic analysis of entire thermal decomposition process of polymeric matrix, total reaction rate of complex solid-state reactions was expressed as the sum of five individual kinetic processes according to a scheme of successive reactions [75,76,77,78]:

(21) $$\frac{d\alpha }{\mathrm{dt}}=\overset{r}{\underset{i=1}{\sum }}c_{i}\frac{{d\alpha }_{i}}{\mathrm{dt}}$$

where r is the number of individual reactions and $c_{i}$ is the weighting coefficient representing the contribution factor of each reaction i. Likewise, weighting coefficients were subject to two restrictions: $c_{i}\geq 0$ and $\sum c_{i}=1$.

Parameters for the proposed scheme,${\;A}_{i}$, $E_{\mathrm{ai}}$,$\;f\left(\alpha_{i}\right),$ and $c_{i}$ were refined using a nonlinear least-squares technique. The objective function (OF) used was defined by the following expression:

(22) $$\mathrm{OF}=\min\overset{N}{\underset{j=1}{\sum }}{\left[{\left(\frac{d\alpha }{\mathrm{dt}}\right)}_{\exp}-{\left(\frac{d\alpha }{\mathrm{dt}}\right)}_{\mathrm{cal}}\right]}^{2}$$

where ${\left(\frac{d\alpha }{\mathrm{dt}}\right)}_{\exp}$ and ${\left(\frac{d\alpha }{\mathrm{dt}}\right)}_{\mathrm{cal}}$ represent experimental and calculated conversion rates, respectively, j refers to a point in the experiment, and N is the total number of experimental points.

**Figure 4:**
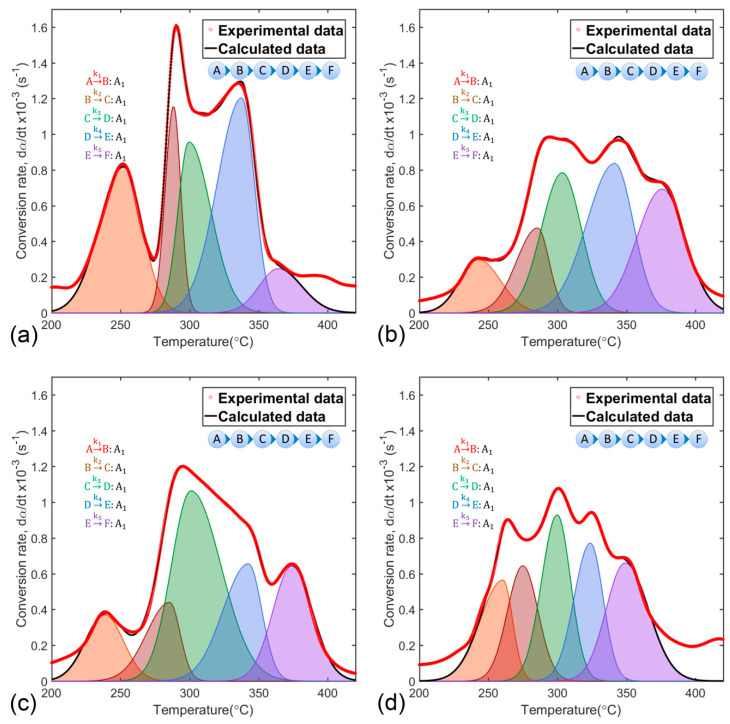
Experimental and calculated conversion rate curves based on a scheme of five successive reactions of polymeric matrix decomposition of the synthesis precursors (**a**) SP, (**b**) SPMg-1, (**c**) SPMg-2, and (**d**) SPMg-3.

Solution to the kinetic problem posed in Equation (21) was achieved by fit convergence between experimental conversion rate curves (red, dotted line) and calculated curves (black line) composed of five separate stages of reaction (shaded area curves), as shown in Figure 4.

Adjustment results validated the scheme of successive reactions as the sequence of processes representing polymeric matrix thermal decomposition. Thus, complex solid-state reactions would result from linear combination of individual data $\left(\frac{{d\alpha }_{i}}{\mathrm{dt}}\right)$ and would be represented by the scheme formed by the Equations (23) to (27).

- Dehydroxylation reaction:
  (23) $$A\overset{k_{1}}{\to }B+H_{2}O↑$$
- Decarboxylation reaction:
  (24) $$B\overset{k_{2}}{\to }C+{\mathrm{CO}}_{2}↑$$
- Multistage combustion reaction:
  (25) $$C\overset{k_{3}}{\to }D+{\mathrm{CO}}_{2}↑$$
  (26) $$D\overset{k_{4}}{\to }E+{\mathrm{CO}}_{2}↑$$
  (27) $$E\overset{k_{5}}{\to }F+{\mathrm{CO}}_{2}↑$$
  where A is the dry synthesis precursor after dehydration; B, C, D, and E are solid intermediate products; F is the final solid product; and $k_{i}$ is reaction rate constant (subscripts i from 1 to 5 correspond to reactions 23 to 27, respectively).

First-order Avrami-Erofeev equation, n = 1, which considers a mechanism of random nucleation followed by an instantaneous growth of nuclei [54,56,79,80], was the kinetic model function f(α), which fitted each of the reactions of the polymeric matrix decomposition process.

Avrami-Erofeev general equation of nucleation and nuclei growth:

(28) $$A_{n}=n\left(1-\alpha \right){\left[-\ln\left(1-\alpha \right)\right]}^{\frac{n-1}{n}}$$

For n = 1:

(29) $$A_{1}=\left(1-\alpha \right)$$

Arrhenius parameters ($E_{a}$ and log(A)), contribution factors ($c_{i}$), peak temperatures ($T_{p}$), and correlation coefficients ($R^{2}$) obtained from the fit procedure based on a scheme of five successive reactions were listed in Table 3.

**Figure 5:**
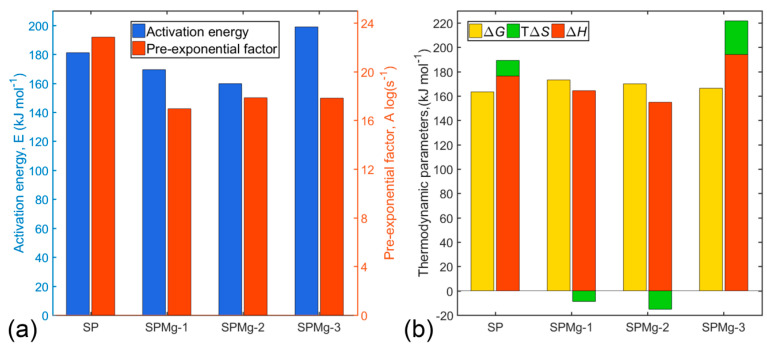
Average values for (**a**) $E_{a}$ and log(A), and (**b**) ${\Delta G}^{\neq },{\;T}_{p}{\Delta S}^{\neq }{\;\mathrm{and}\;\Delta H}^{\neq }\;$ from polymer matrix thermal decomposition process of synthesis precursors prepared with different Mg doping concentrations.

Average values for $E_{a}$ and log(A) were calculated taking into account contribution of each of the reaction stages and are illustrated in Figure 5a according to their magnesium doping composition.

High values of $E_{a}$ and log(A) might be related to high mass losses and, consequently, to lower CO_2_ sorption, as is the case of SP and SPMg-3 precursors, whereas low values might be related to lower mass losses due to higher CO_2_ sorption, as is the case of SPMg-2 and SPMg-1 precursors.

Thus, existence of an inverse relationship between Arrhenius parameters and mass retention, as a result of CO_2_ sorption, which is responsible for carbonates’ formation in intermediate phases, would agree with the order of mass loss of precursors in the third thermal decomposition zone (SP > SPM-3 > SPMg-1 > SPMg-2), as shown in Figure 2, and would indicate that high values of $E_{a}$ and log(A) are related to the formation of thermodynamically stable spinel phases in an organic content post-combustion stage with a lower energy requirement for decomposition of intermediate phases through CO_2_ desorption.

The main impact of magnesium doping on thermal decomposition kinetics was elucidated between SP and SPMg-3 precursors. Where increase of $E_{a}$ and decrease of log(A) observed in SPMg-3 precursor, compared to SP precursor, would demonstrate that magnesium incorporation, as a doping agent, reduces reaction rates of polymeric matrix decomposition because it favors a greater CO_2_ sorption.

### 3.4. Transition State Thermodynamic Parameters of Polymeric Matrix Thermal Decomposition

Thermodynamic parameters such as the changes of the enthalpy, Gibbs’ free energy, and entropy for the activated complex were obtained by combining Arrhenius equations (Equation (2)) and Eyring. The latter, derived from transition state theory (activated complex) [81], represents constant rate of a chemical reaction according to the following expression [82,83,84]:

(30) $$k=\kappa \frac{k_{B}T_{p}}{h}\exp\left(\frac{{\Delta S}^{\neq }}{R}\right)\exp\left(-\frac{{\Delta H}^{\neq }}{\mathrm{RT}}\right)$$

where κ is the transmission coefficient and can be considered close to unity, ${\Delta S}^{\neq }$ and ${\Delta H}^{\neq }$ are the entropy and enthalpy changes of the activated complex, respectively, $k_{B}$ is Boltzmann’s constant (1.381 × 10^−23^ J K^−1^), h is Plank’s constant (6.626 × 10^−34^ J s), and $T_{p}$ is the peak temperature on a DTG curve.

Entropy change, linked to the pre-exponential factor, was calculated according to the formula:

(31) $${\Delta S}^{\neq }=\mathrm{Rln}\left(\frac{\mathrm{Ah}}{{\kappa \mathrm{ek}}_{B}T_{p}}\right)$$

where e is Euler’s number (2.7183).

Enthalpy change was determined by activation energy with the expression:

(32) $${\Delta H}^{\neq }=E-{\mathrm{RT}}_{p}$$

Finally, Gibbs’ free energy change in active complex formation from reagents was calculated using the well-known thermodynamic equation:

(33) $${\Delta G}^{\neq }={\Delta H}^{\neq }-T_{p}{\Delta S}^{\neq }$$

Values for ${\Delta S}^{\neq }$, ${\Delta H}^{\neq }$, and ${\Delta G}^{\neq }$, listed in Table 4, were calculated at temperature peaks $T_{\mathrm{pi}}$, because these temperatures characterize the highest rate of each of five polymeric matrix thermal decomposition reactions. Average values of thermodynamic parameters were illustrated as shown in Figure 5b according to magnesium doping concentration.

Positive ${\Delta G}^{\neq }$ and ${\Delta H}^{\neq }$ values for all precursors showed that formation of activated complexes, in each of the polymeric matrix decomposition reactions, corresponds to nonspontaneous processes involving introduction of heat.

As shown in Figure 5b, entropy values for SP and SPMg-3 precursors are positive, while for SPMg-1 and SPMg-2 precursors they are negative. These might mean that activated complexes of SP and SPMg-3 precursors were less structured or organized compared to initial reagents, which would indicate that formation of active complexes would be accompanied by an increase in total system entropy (${\Delta S}^{\neq }>0$). In contrast, formation of activated complexes of SPMg-1 and SPMg-2 precursors would be related to a decrease in entropy (${\Delta S}^{\neq }<0$). Therefore, in terms of transition state theory or activated complex, polymeric matrix thermal decomposition reactions can be interpreted as “fast” for SP and SPMg-3 precursors, which favor formation of spinel phases, and “slow” for SPMg-1 and SPMg-2 precursors, which result in formation of intermediate oxide-carbonate phases due to a high CO_2_ sorption.

### 3.5. Stoichiometric, Structural, and Morphological Analysis

According to results obtained from TG analysis and effect of Mg concentration on kinetics and thermodynamics of synthesis precursors’ thermal decomposition, an optimal two-stage heat treatment program was applied to prepare oxide powders with LiMg_x_Mn_2−x_O_4_ (x = 0.00, 0.02, 0.05, 0.10) spinel phase: A first stage of calcination in atmospheric air at 500 °C for 4 h to ensure complete combustion of precursors’ polymeric matrix with negligible CO_2_ sorption and a second stage to desorb CO_2_ residues and sinter spinel oxide powders in air atmosphere at 750 °C for 12 h.

Li/Mn/Mg cation ratios for LiMg_x_Mn_2−x_O_4_ nanomaterials measured by AAS are given in Table 5. Cationic composition results revealed that conditions established in the heat treatment program partially modified stoichiometry of spinels, allowing to preserve a fraction of the excess Li used in precursors’ preparation, which reduced molar ratio and increased average valence of Mn above its corresponding nominal composition.

**Figure 6:**
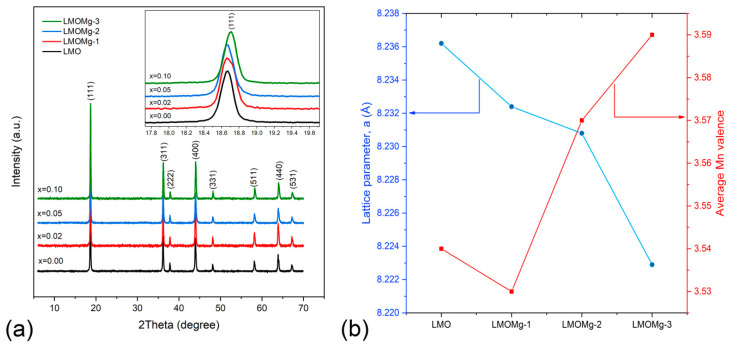
(**a**) LiMg_x_Mn_2−x_O_4_ (x = 0.00, 0.02, 0.05, 0.10) nanometer powders’ diffraction patterns. Inset shows displacement of peak (111) towards higher angles with increase of x. (**b**) Decrease of cell parameter “a” and raised average Mn valence with increasing doping concentration of Mg.

Figure 6 shows XRD patterns of LiMg_x_Mn_2−x_O_4_ (x = 0.00, 0.02, 0.05, 0.10) samples. All diffraction peaks (111), (311), (222), (400), (331), (511), (440), and (531) show good consistency with standard LiMn_2_O_4_ diffraction peaks from International Centre for Diffraction Data (ICDD No. 00–035–0782), respectively.

This indicates that LiMg_x_Mn_2−x_O_4_ samples possess the cubic spinel structure with $\mathrm{Fd}\bar{3}m$ space group of LiMn_2_O_4_, suggesting that Mg^2+^ doping does not change the intrinsic cubic symmetry of spinel structure. No impurities such as L_2_MnO_3_, LiMnO_2_, Mn_2_O_3,_ or MnOχ were detected, which provides evidence of high purity of synthesized products. In addition, sharpness of the main peaks (111), (311), and (400) indicates that samples have high crystallinity.

On the other hand, the diffraction peak for the lattice plane (220) (2θ ≈ 30.9°), which arises only from diffraction of tetrahedral sites (8a) and could not be observed in an LMO XRD pattern due to low scattering ability of lithium atoms, was not observed in diffraction patterns of LiMg_x_Mn_2−x_O_4_ (x = 0.00, 0.02, 0.05, 0.10) samples in Figure 6, which suggests that Mg^2+^ ions used in doping only occupy octahedral sites (16d) in substitution of manganese ions [18,24].

**Figure 7:**
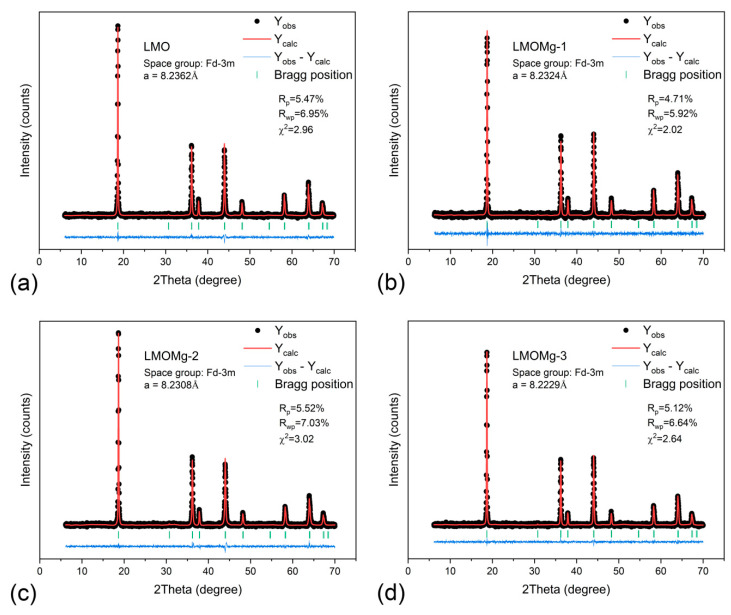
Rietveld refinement results of powder X-ray diffraction (XRD) data from magnesium-doped manganese spinels: (**a**) LMO, (**b**) LMOMg-1, (**c**) LMOMg-2, and (**d**) LMOMg-3. Data points are shown as black dots. The calculated pattern is shown with a solid red line. Vertical green bars show Bragg peak positions. A blue line at the bottom shows the difference between data and calculated intensities.

Refinement of synthetized spinels was performed using eight well-defined diffraction lines with indexing in a $\mathrm{Fd}\bar{3}m$ space group cubic system. Figure 7 shows Rietveld refinement results. Calculated unit cell parameters are listed in Table 6.

A gradual decrease in lattice parameter was observed with increasing magnesium concentration “x”. This is due to differences in ion radius between Mn^3+^ (0.645 Å) and Mn^4+^ (0.53 Å) ions. When Mg^2+^ is doped at Mn^3+^ site of the spinel structure, the number of Mn^4+^ ions increases in order to maintain charge balance condition resulting in an increase in average Mn valence (Figure 6b), which suppresses Jahn-Teller distortion [85,86]. Therefore, as shown in Figure 6b, lattice parameter “a” of the unit cell decreased with increasing magnesium content. This behavior was also manifested as a slight shift of peaks toward higher angles, as can be seen in the inset within Figure 6a. Lattice contraction due to substitution with Mg ion was consistent with results previously reported by Subramania et al. [87].

The atom location confused degree, which is closely related to the electrochemical properties of the LMO and represents the exchange of atomic sites between lithium and manganese ions that lead to the formation of an anti-spinel structure [88,89], was analyzed through the intensity ratio between the I(311)/I(400) peaks. In Table 6, values obtained for the I(311)/I(400) ratio were close to a theoretical value of 1.86, corresponding to a LMO spinel without exchange of atomic sites [90], which would indicate that LiMg_x_Mn_2−x_O_4_ samples have an insignificant degree of confusion and, therefore, would imply high structural stability of the Mn_2_O_4_ spinel framework.

Scherrer’s formula (Equation (34)) [91,92] and full width at half maximum (FWHM) for the main diffraction peaks (111), (311), and (400) were used to describe crystallite size variation in spinels prepared with magnesium doping. Obtained data were summarized as listed in Table 6.

(34) $$\mathrm{Dc}=\frac{k\lambda }{\beta \cos\theta }$$

where crystallite size is denoted by Dc, shape factor or Scherrer constant k is approximately 0.94 for spherical crystals with cubic symmetry, λ is the average wavelength of the X-ray beam used (CuKα, λ = 1.54178 Å), β is FWHM of the highest intensity peaks, and θ is Bragg angle in radians.

Results showed that nondoped magnesium spinel had the largest average crystallite size, while for magnesium-doped spinels, average crystallite size increased with increasing doping concentration. This behavior reflected existence of an inverse relationship between nucleation rate and crystallite growth with thermal inertia increase, caused by CO_2_ chemisorption as a result of addition of magnesium. In this way, crystallite size would reflect the amount of energy consumed in CO_2_ desorption and formation of a thermodynamically stable spinel phase, i.e., small crystallites are resulting from higher energy consumption and vice versa for large crystallites.

**Figure 8:**
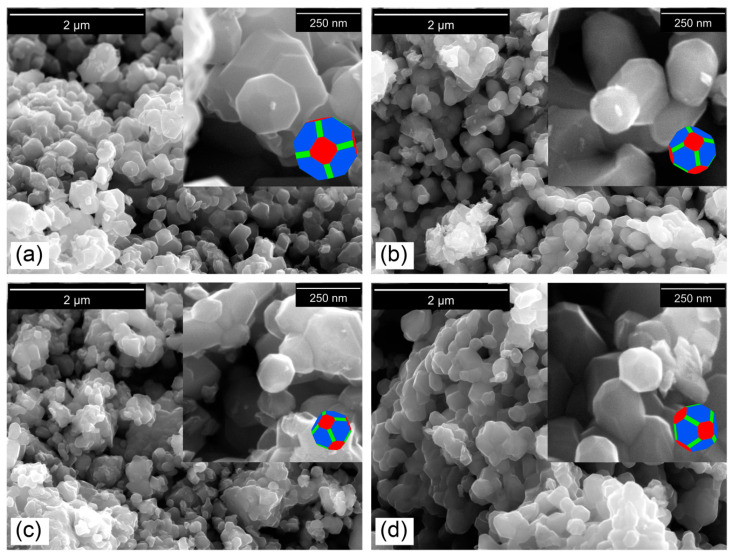
FE-SEM images of the nanometric powders of magnesium-doped manganese spinels: (**a**) LMO, (**b**) LMOMg-1, (**c**) LMOMg-2, and (**d**) LMOMg-3. Insets show truncated octahedral-type morphology of primary particles that make up spinel powders. The (111) planes in blue, (100) planes in red, and (110) planes in green.

Morphology of synthesized nanocrystalline powders was observed in the micrographs shown in Figure 8. LiMg_x_Mn_2−x_O_4_ spinel samples presented a truncated octahedral morphology (insets within Figure 8), which contains mainly truncated planes (111) with a small portion of (100) and (110) planes. The (111) faces possess lowest surface energy, a most dense arrangement of Mn atoms, and can form a stable layer at solid electrolyte interface and mitigate Mn dissolution, thus improving cycle stability, while (100) and (110) truncated faces, with less dense arrangement of ions, are aligned to Li^+^ diffusion channels, therefore increasing discharge capacity and facilitating rate capabilities [93,94,95].

On the other hand, as shown in Figure 8, primary particles would be in a slightly agglomerated state, which could be beneficial to provide a good material-packing density leading to higher bulk capacity.

**Figure 9:**
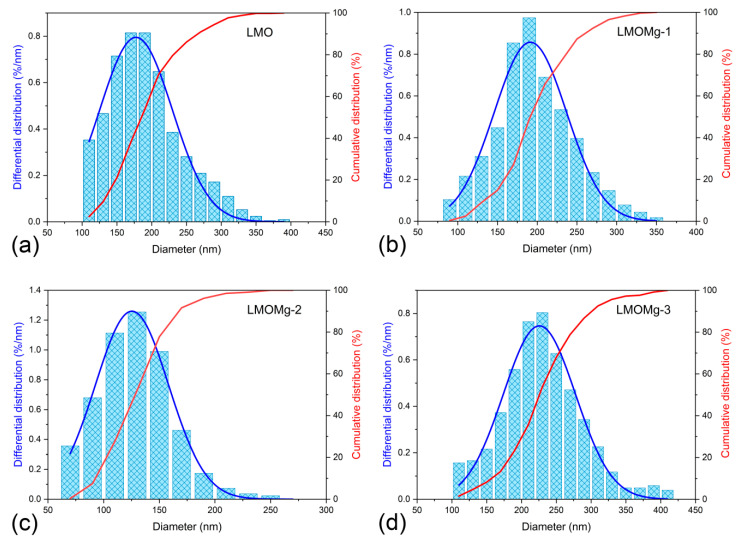
Differential and cumulative distribution of particle size of the nanometric powders of magnesium-doped manganese spinels: (**a**) LMO, (**b**) LMOMg-1, (**c**) LMOMg-2, and (**d**) LMOMg-3.

The particle size distribution was determined by measuring the equivalent circle diameter of the primary particles. The resulting values were plotted on histograms and cumulative curves as shown in Figure 9. The frequency distribution was adjusted with a Gaussian function and the results on average diameters of nanoparticles are summarized in Table 7.

The variation of the mean diameter of the primary particles with the magnesium doping concentration was consistent with the kinetic and thermodynamic parameters obtained, because large particle sizes are related to fast reactions and with lower energy consumption for spinel phase formation.

## 4. Conclusions

Through mass-loss profiles of synthesis precursors, four thermal decomposition zones were defined: (1) Dehydration, (2) polymeric matrix decomposition, (3) carbonate decomposition and formation of manganese oxide spinel, and (4) manganese oxide spinel decomposition. Thermal decomposition of polymeric matrix was identified as the main zone that encompassed fundamental reactions initiating formation of a thermodynamically stable LiMn_2_O_4_ spinel phase.

In the polymeric matrix decomposition zone, magnesium incorporation favored CO_2_ chemisorption reaction and decreased the thermal conductivity of synthesis precursors. Inverse variation of thermal inertia with magnesium concentration caused a displacement of conversion curves at high temperatures and a decrease in conversion rate.

The nonlinear least-squares technique was applied to determine kinetic triplets, ($E_{a}$, A, and f(α)), by adjusting conversion rate profiles with a scheme of five successive reactions. The first-order Avrami-Erofeev equation was the reaction model that represented the polymeric matrix decomposition zone, and high average Arrhenius parameters values ($E_{a}$ and A) would be related to low CO_2_ sorption and lower energy requirement for pure spinel phase formation with and without magnesium doping.

Thermodynamic functions (${\Delta S}^{\neq }$, ${\Delta G}^{\neq },$ and ${\Delta H}^{\neq }$) revealed that activated complexes’ formation would be associated with nonspontaneous endothermic processes. Evaluation of ${\Delta S}^{\neq }$ revealed that thermal decomposition of the SPMg-1 and SPMg-2 precursors with low magnesium content would be associated with slow reactions (${\Delta S}^{\neq }$ < 0), while fast reactions would be related to the SP and SPMg-3 precursors without doping and with higher magnesium concentration, respectively.

From analysis of kinetic and thermodynamic results, it was established that CO_2_ desorption was the limiting step in formation of thermodynamically stable spinel phases with and without magnesium doping.

Based on TG experiments and the effect of magnesium on kinetics and thermodynamics of thermal decomposition of synthesis precursors, a two-stage heat treatment program was determined for synthesis of LiMg_x_Mn_2−x_O_4_ (x = 0.00, 0.02, 0.05, 0.10): Calcination at 500 °C for 4 h and sintering at 750 °C for 12 h. The result was a series of nanometric powders, in a mean diameter range of 125.4 ± 32.9 nm to 225.7 ± 51.8 nm of pure phase with truncated octahedral morphology and cubic structure of LiMn_2_O_4_ spinel with $\mathrm{Fd}\bar{3}m$ space group.

Heat treatment conditions partially modified stoichiometry of the prepared spinels due to retention of an excess lithium fraction, which decreased manganese molar ratio but increased its average oxidation state.

Replacement of Mn^3+^ by Mg^2+^ did not change the cubic spinel structure but caused a unit cell shrinkage, where cell parameter “a” decreased in inverse proportion to an increase in “x”.

Growth rate of the primary particles of spinel powder was affected by magnesium concentration and, consequently, by the amount of CO_2_ chemisorption. Thus, large crystals were formed from precursors with lower CO_2_ sorption and vice versa for small crystals.

The method developed for kinetic and thermodynamic analysis proved to be a powerful tool for obtaining theoretical information useful for determining optimal heat treatment parameters for preparing high crystallinity, structural stability, and homogeneous morphology of LiMg_x_Mn_2−x_O_4_ compounds. All these characteristics make them promising cathode materials for lithium ion batteries. A deep study of their electrochemical properties is planned to be carried out in future work.

## Figures and Tables

**Table 1:** Percentages and maximum rates of mass loss of the thermal decomposition zones of the synthesis precursors.

		Stage 1	Stage 2	Stage 3	Stage 4
Sample	Temperature range	25 °C–200 °C	200 °C–420 °C	420 °C–790 °C	790 °C–950 °C
SP x = 0.00	Mass loss (%)	2.19	46.02	8.51	1.61
	Max. decomposition rate (% s^−1^)	0.67 × 10^−2^	9.37 × 10^−2^	1.22 × 10^−2^	0.6 × 10^−2^
SPMg-1 x = 0.02	Mass loss (%)	3.35	40.65	6.6	2.58
	Max. decomposition rate (% s^−1^)	0.53 × 10^−2^	5.24 × 10^−2^	0.87 × 10^−2^	0.73 × 10^−2^
SPMg-2 x = 0.05	Mass loss (%)	3.13	38.2	4.94	3.69
	Max. decomposition rate (% s^−1^)	0.61 × 10^−2^	6 × 10^−2^	0.5 × 10^−2^	0.76 × 10^−2^
SPMg-3 x = 0.10	Mass loss (%)	6.45	36.61	9.16	2.31
	Max. decomposition rate (% s^−1^)	1.05 × 10^−2^	5.89 × 10^−2^	1.29 × 10^−2^	0.71 × 10^−2^

**Table 2:** Reaction models and kinetic parameters of each single reaction of polymeric matrix decomposition process of precursors with different magnesium doping concentrations.

Synthesis Precursor	Peak Number	Reaction Model	$E_{a}\;(\mathrm{kJ}\;{\mathrm{mol}}^{-1})$	log(A)
SP x = 0.00	1	Avrami–Erofeev equation, n = 1	157.68	13.77
	2		249.19	21.72
	3		133.79	12.80
	4		189.41	14.56
	5		257.35	18.78
SPMg-1 x = 0.02	1	Avrami–Erofeev equation, n = 1	135.66	11.95
	2		187.88	15.43
	3		170.99	14.67
	4		157.76	12.44
	5		180.74	13.65
SPMg-2 x = 0.05	1	Avrami–Erofeev equation, n = 1	123.54	11.56
	2		206.82	17.86
	3		101.50	6.95
	4		261.91	20.42
	5		206.18	14.63
SPMg-3 x = 0.10	1	Avrami–Erofeev equation, n = 1	209.10	18.09
	2		148.30	11.91
	3		198.30	16.33
	4		194.48	15.21
	5		181.01	12.44

**Table 3:** Kinetic parameters, contribution factors, peak temperatures, and correlation coefficients from fit of experimental conversion rates of polymeric matrix thermal decomposition with a scheme of five successive reactions.

Synthesis Precursor	Peak Number	$c_{i}$	$T_{p}\;({}^{\circ}C)$	$E_{a}\;(\mathrm{kJ}\;{\mathrm{mol}}^{-1})$	log(A)	$R^{2}$
SP x = 0.00	1	0.23	251.94	160.57	14.07	0.98
	2	0.12	288.36	265.02	23.75	
	3	0.23	299.17	129.57	9.90	
	4	0.30	330.75	181.37	13.78	
	5	0.12	343.27	233.96	18.07	
	Total	1.00	Average	181.13	22.84	
SPMg-1 x = 0.02	1	0.07	242.99	142.64	12.44	0.99
	2	0.15	285.67	209.40	17.78	
	3	0.21	303.59	165.53	13.04	
	4	0.30	341.46	159.56	11.59	
	5	0.27	375.87	168.39	11.48	
	Total	1.00	Average	169.41	16.96	
SPMg-2 x = 0.05	1	0.10	238.71	149.12	13.24	0.99
	2	0.13	285.52	213.13	18.13	
	3	0.43	300.99	94.02	6.30	
	4	0.14	342.55	240.03	18.61	
	5	0.21	374.04	215.91	15.44	
	Total	1.00	Average	159.77	17.86	
SPMg-3 x = 0.10	1	0.14	260.59	207.85	18.70	0.92
	2	0.14	275.00	154.45	12.86	
	3	0.24	299.86	211.79	17.53	
	4	0.23	323.86	218.74	17.33	
	5	0.26	348.98	189.34	13.87	
	Total	1.00	Average	198.92	17.91	

**Table 4:** $\mathrm{The}\;{\Delta S}^{\neq }$, ${\Delta H}^{\neq }$, and ${\Delta G}^{\neq }$ values for formation of activated complexes in the polymeric matrix thermal decomposition zone of doped synthesis precursors with different concentrations of magnesium.

Synthesis Precursor	Peak Number	$c_{i}$	$T_{p}\;(K)$	$\Delta S^{\neq }$ (J mol^−1^ K^−1^)	$\Delta H^{\neq }$ (kJ mol^−1^)	$\Delta G^{\neq }$ (kJ mol^−1^)
SP x = 0.00	1	0.23	525.09	11.40	156.21	150.22
	2	0.12	561.51	196.19	260.35	150.18
	3	0.23	572.32	−69.07	124.81	164.34
	4	0.30	603.90	4.69	176.35	173.51
	5	0.12	616.42	86.63	228.83	175.44
	Total	1.00	Average	22.40	176.35	163.42
SPMg-1 x = 0.02	1	0.07	516.14	−19.74	138.35	148.53
	2	0.15	558.82	81.91	204.75	158.98
	3	0.21	576.74	−9.02	160.74	165.94
	4	0.30	614.61	−37.46	154.45	177.48
	5	0.27	649.02	−39.85	163.00	188.86
	Total	1.00	Average	−12.91	164.42	173.19
SPMg-2 x = 0.05	1	0.10	511.86	−4.31	144.86	147.07
	2	0.13	558.67	88.63	208.49	158.97
	3	0.43	574.14	−138.01	89.25	168.48
	4	0.14	615.70	96.92	234.91	175.24
	5	0.21	647.19	35.91	210.53	187.29
	Total	1.00	Average	−28.04	154.88	170.04
SPMg-3 x = 0.10	1	0.14	533.74	99.92	203.41	150.08
	2	0.14	548.15	−12.04	149.89	156.49
	3	0.24	573.01	76.97	207.03	162.92
	4	0.23	597.01	72.84	213.78	170.29
	5	0.26	622.13	6.18	184.17	180.32
	Total	1.00	Average	48.39	194.08	166.37

**Table 5:** LiMg_x_Mn_2−x_O_4_ (x = 0.00, 0.02, 0.05, 0.10) chemical composition as determined by AAS and the average Mn valence.

Sample Name	Nominal		Experimental	
	Stoichiometry	Average Mn Valence	Stoichiometry	Average Mn Valence
LMO	LiMn_2_O_4_	3.50	Li_1.03_Mn_1.97_O_4_	3.54
LMOMg-1	LiMg_0.02_Mn_1.98_O_4_	3.52	Li_1.01_Mg_0.02_Mn_1.97_O_4_	3.53
LMOMg-2	LiMg_0.05_Mn_1.95_O_4_	3.54	Li_1.03_Mg_0.05_Mn_1.92_O_4_	3.57
LMOMg-3	LiMg_0.10_Mn_1.90_O_4_	3.58	Li_1.01_Mg_0.10_Mn_1.89_O_4_	3.59

**Table 6:** Rietveld refinement, crystal parameters, and crystallite size for LiMg_x_Mn_2−x_O_4_ (x = 0.00, 0.02, 0.05, 0.10) samples.

Sample Name	LMO	LMOMg-1	LMOMg-2	LMOMg-3
Symmetry	cubic			
Space group	$\mathrm{Fd}\bar{3}m$			
Lattice parameter (Å)	8.2362	8.2324	8.2308	8.2229
Unit cell volume (Å^3^)	558.6968	557.9358	557.6075	556.0020
R_p_ (%)	5.47	4.71	5.52	5.12
R_wp_ (%)	6.95	5.92	7.03	6.64
χ^2^	2.96	2.02	3.02	2.64
I(311)/I(400)	0.87	0.82	0.87	0.83
FWHM_111_ (°)	0.148	0.170	0.152	0.146
FWHM_311_ (°)	0.155	0.173	0.171	0.167
FWHM_400_ (°)	0.159	0.175	0.183	0.178
Dc Average (nm)	56.51	50.47	51.91	53.45

**Table 7:** Results of statistical analysis of the particle size distribution for LiMg_x_Mn_2−x_O_4_ (x = 0.00, 0.02, 0.05, 0.10) samples.

Sample	LMO	LMOMg-1	LMOMg-2	LMOMg-3
Mean diameter (nm)	177.5	191.4	125.4	225.7
Standard Deviation (nm)	52.3	45.7	32.9	51.8
